# Memantine for Mitigation of Neurocognitive Toxicity Following Radiation to the Brain

**DOI:** 10.1200/GO.20.00551

**Published:** 2021-01-06

**Authors:** Danielle A. Cunningham, Paul D. Brown, Nadia N. I. Laack

**Affiliations:** Danielle A. Cunningham, MD; Paul D. Brown, MD; and Nadia N. I. Laack, MD, Department of Radiation Oncology, Mayo Clinic, Rochester, MN

## TO THE EDITOR:

Chilukuri et al^1^ have composed an excellent review of pharmacologic interventions for reduction of neurocognitive deficits following radiation to the brain. We strongly agree with the conclusions of the authors that memantine is a safe, effective, accessible, and relatively inexpensive intervention that serves to improve cognitive function following radiation to the brain.

We hope to add clarification to the authors' interpretation of RTOG 0614.^2^ Although it is true that statistical significance was not reached at 24 weeks (*P* = .059) for decline in delayed recall, it is important to note that only 149 of 554 patients were analyzable at that time point, resulting in 35% statistical power to detect an absolute 0.87 difference. Additionally, the authors describe a small decline in delayed recall in the memantine arm at 24 weeks, although in actuality the patients on the memantine arm did not demonstrate cognitive decline on the Hopkins Verbal Learning Test-Revised (HVLT-R) Delayed Recognition (median decline of 0) compared with the placebo arm (median decline of −0.90) at 24 weeks.

On RTOG 0614, time to cognitive failure, defined as the first failure on any administered neurocognitive tests or a 2-SD decline from baseline for any test, significantly favored the memantine arm (HR, 0.87; 95% CI, 0.62 to 0.99; *P* = .01), with a probability of cognitive function failure at 24 weeks demonstrating a 21% relative reduction with memantine. We emphasize that time to cognitive failure is an important end point for patients with brain metastases as the expected survival is short and may demonstrate a superior neurocognition correlate when compared with a landmark analysis at 24 weeks. On RTOG 0614, the median overall survival was 6 months (26 weeks) and was not different between arms.

We also hope to amend that on table 1, memantine was studied in RTOG 0614 for patients with brain metastases only, and patients with primary brain tumors were not included. Additionally, in the toxicities column, it is important to note that the alopecia seen in patients treated on RTOG 0614 was caused by the whole-brain radiation rather than memantine, with no difference in alopecia between patients treated with memantine or placebo.

Memantine is overall very well tolerated, and in double-blind placebo-controlled trials of patients with dementia, the likelihood of discontinuation due to adverse reaction was 11.5% in the placebo group and 10.1% in the memantine cohort, with no significant difference between the groups. The most common adverse reactions to memantine were uncommon, including dizziness (7% memantine *v* 5% placebo), headache (6% memantine *v* 3% placebo), confusion (6% memantine *v* 5% placebo), constipation (5% memantine *v* 3% placebo), and fatigue (2% memantine *v* 1% placebo).^3^

Overall, we agree with the authors that memantine is a useful tool for mitigation of neurocognitive effects of radiation to the brain and add additional strength to their assertion. We agree that further research is needed to determine the benefit of memantine for patients beyond the original whole-brain radiation indication. We are actively recruiting pediatric patients to our study evaluating the impact of memantine on cognition after radiation treatment to the brain for patients of age 4-18 years (ClinicalTrials.gov identifier: NCT04217694) and look forward to results of other studies investigating memantine to improve neurocognitive outcomes.
